# Virtual 3D tumor marking-exact intraoperative coordinate mapping improve post-operative radiotherapy

**DOI:** 10.1186/1748-717X-6-159

**Published:** 2011-11-16

**Authors:** Harald Essig, Majeed Rana, Andreas Meyer, André M Eckardt, Horst Kokemueller, Constantin von See, Daniel Lindhorst, Frank Tavassol, Martin Ruecker, Nils-Claudius Gellrich

**Affiliations:** 1 Department of Oral & Maxillofacial Surgery, Hannover Medical School, Hannover Germany; 2 Department of Radiotherapy, Hannover Medical School, Hannover, Germany

## Abstract

The quality of the interdisciplinary interface in oncological treatment between surgery, pathology and radiotherapy is mainly dependent on reliable anatomical three-dimensional (3D) allocation of specimen and their context sensitive interpretation which defines further treatment protocols. Computer-assisted preoperative planning (CAPP) allows for outlining macroscopical tumor size and margins. A new technique facilitates the 3D virtual marking and mapping of frozen sections and resection margins or important surgical intraoperative information. These data could be stored in DICOM format (Digital Imaging and Communication in Medicine) in terms of augmented reality and transferred to communicate patient's specific tumor information (invasion to vessels and nerves, non-resectable tumor) to oncologists, radiotherapists and pathologists.

## Introduction

Three of the most challenging interfaces in oncologic treatment in head and neck cancer exist between surgeon and pathologist just as between surgeon and radiotherapist and/or oncologist. The former interface is relevant to hopefully confirm the achieved full resection (R0-resection) which is especially difficult due to the complex anatomy of the head and neck region. The recording and naming of frozen sections or resection margins does often not allow for later well-defined three-dimensional (3D) orientation. Due to this 3D-complexity in between written words and the real location pathologists are not able to rule out residual tumor without consultation of the surgeon, who sometimes has to stitch more to his personal memory than to reliable recorded information.

**Figure 1:**
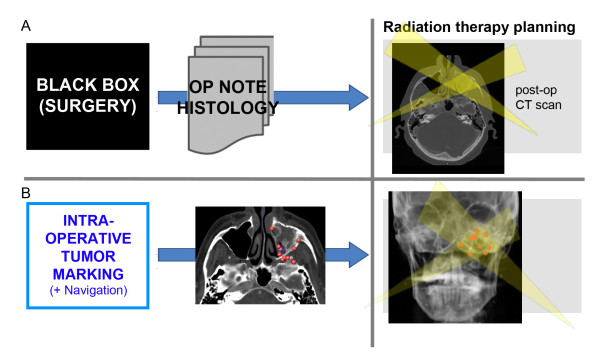
**Conventional interface between surgery and radiotherapy**. (A) Radiotherapist obtains limited information out of OP notes, histology, and post-operative CT scan. (B) Intra-operative markings are transferred to radiotherapists to complete 3D information.

If there is an indication for adjuvant radiation therapy, such as minimal tumor residuals (R1-resection), the same problem discounts for radiation therapy planning to be challenging: the radiotherapist could not gain access to reliable intra-operative information and uses mainly the results of the histological findings, the operation protocol, and the post-operative computed tomography (CT scan) for the simulation planning (Figure 1).

Summing up, histopathological findings should be ideally three-dimensionally mapped and information should be without loss and ideally language-independent digitally stored, to improve the interdisciplinary interface to the benefit of the patient.

Computer-assisted pre-operative planning (CAPP) is commonly used in intra-operative visualization and reconstruction in ablative surgery of the head and neck [1]. Therefore multimodal three-dimensional imaging could be matched to outline tumor dimensions and demonstrate virtually augmented surgical margins. The minor additional expenses to enable intra-operative navigation ease anatomical orientation and true-to-original reconstruction after ablative surgery [2-5].

Marking with clips and different dyes is published in literature [6-9], but virtual marking and mapping is a new technique that allows for intra-operative marking of locations where specimen, for instance frozen sections or resection margins, are taken. These data could be saved in DICOM-format (Digital Imaging and Communication in Medicine) and transferred to pathologists and radiotherapists.

The workflow of virtual 3D marking including preparation, intraoperative setting and postoperative postprocessing is described and illustrated.

## Materials and methods

In patients with malignant head and neck tumors located at or extended to the skull base, to the orbit or to the paranasal sinuses, resection may present a significant challenge because of critical vascular and neural structures and the close relation to the brain. To maintain a balance between aggressive surgical approaches to achieve curative resection (R0-resection) and obtaining relevant quality of life is a difficult task. Therefore limited surgical treatment (R1-resection) is often combined with adjuvant radiation therapy.

**Figure 2:**
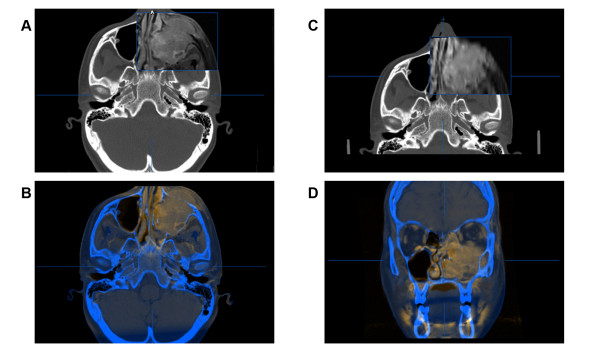
**Fusion of initial CT scan data and MRI**. (A+C) Window shows part of the MRI overlapping the initial CT scan. Both image modalities are shown simultaneously in transversal (B) and coronal (D) view.

Clinical diagnostics include often computed tomography (CT) and magnetic resonance imaging (MRI) to visualize the extent of the tumor and its destruction area (Figure 2). Additional findings complete the tumor staging and allow for adequate treatment planning. Among the advantages of computer-assisted surgery (CAS) in skull base surgery are firstly the three-dimensional intraoperative visualization, secondly the segmentation of vital structures to limit resection (danger zone) and thirdly the planning of the reconstruction [1]. The novel planning and visualization platform iPlan 3.0 (Brainlab^®^, Feldkirchen, Germany) supports intraoperative virtual marking and, for the first time, allows for the export of these collected data (point cloud) into standard DICOM-format.

Postprocessing is necessary to label the virtual markings with the result of the final histological findings. Only the tumor residuals confirmed by the pathologist are included into the initial CT data set. The Houndsfield value of these voxels are determined outside the range of the original data set to illustrate the manipulation.

The workflow is now descriped in detail and can be divided into the following categories:

1. Preparation for intraoperative navigation

2. Intraoperative navigation

3. Postprocessing of the data set

4. Export of the data set

5. Import into the radiation therapy simulation platform

### Preparation for intraoperative Navigation

**Figure 3:**
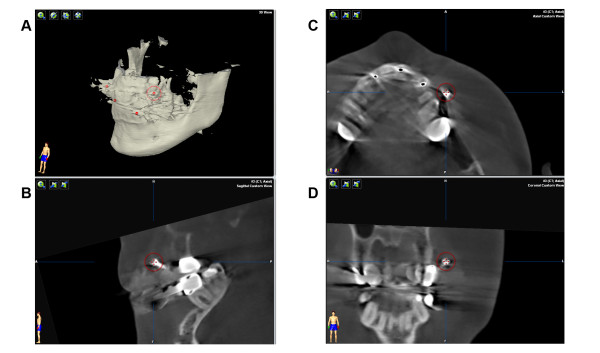
**3D image for tumor diagnostic**. To enable Computer-assisted surgery, tumor diagnostic 3D imaging has to be complemented with a marked 3D data set. The authors recommend a cone beam CT scan subvolume with included navigation splint.

**Figure 4:**
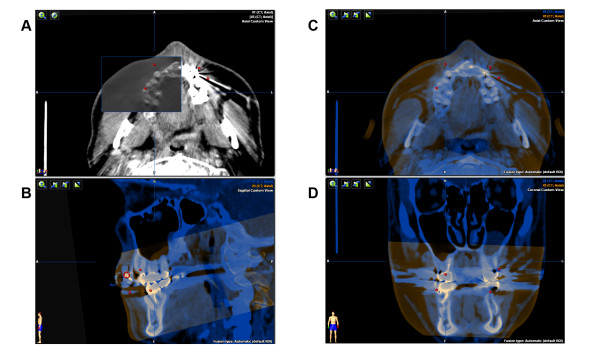
**Superimposing of the subvolume**. Superimposing of the subvolume with the initial CT scan (demonstrated in different planes A-D).

Prior to skull base surgery radiological findings are performed and include in the majority of cases a CT scan for the diagnostics of bony destruction and the potential involvement of the meninges as well as a MRI scan for the soft tissue extent of the tumor. Both modalities could be used for intraoperative visualization but the authors consider the CT scan as indispensable for navigation. There are two possibilities to make the CT scan suitable for navigation. The best option is to use navigation landmarks during the acquisition of the CT scan such as four 2.0 cross-drive titanium-miniscrews (Synthes^®^) or a dental splint with four screw markers. If there is already a pre-existing CT scan, the authors recommend rather a cone beam CT scan (CBCT) (Figure 3) than an additional CT scan after inserting the navigation landmarks to reduce radiation dose for the patient [10]. All different modalities are imported into the planning platform (iPlan 3.0, Brainlab^®^, Feldkirchen, Germany) and image fusion allows for ideal analysis of the patient's pathology (Figure 4). The tumor extent could be outlined manually using different tools, like brushing and autosegmentation. The result of this tumor segmentation could be virtually augmented to demonstrate the extent of the required resection (in many tumor entities at least 1 cm in every dimension). Vital structures such as the carotid arteries and important cranial nerves could be segmented and relation to the augmented tumor segmentation could illustrate of the ablative surgery. For reconstruction virtual planning is performed mainly by using mirrored templates of the non-affected side.

### Intraoperative Navigation

The fusion of the different image modalities is part of the preoperative planning and is available during the intraoperative navigation. A "skull reference base" with three trackable spheres is inserted into the patient's skull, to identify the 3D position and orientation of the patient. The included landmarks of the data sets, either via navigation splint or screw markers, enable the matching of the virtual data set (CT, MRI, CBCT scan) with the real patient's anatomy using the infrared-based navigation system (Kolibri-Brainlab^®^). This procedure is called registration and could be redone if the navigation shows non-acceptable discrepancies. The usual overall root mean error (RMS) should be less than 0.5 to 0.8 mm.

Pointers and tracked instruments are displayed in real-time. Different planes (coronal, axial, and sagittal view) and a 3D reconstruction are available. Vital structures which are segmented could be visualized and thus protected.

By recording intra-operative landmarks (acquired points) locations of biopsies, frozen sections, and surgical margins could be marked. These points could be named additionally. In the everyday practice the specimen were anatomically named and sequentially numbered.

### Postprocessing of the data set

**Figure 5:**
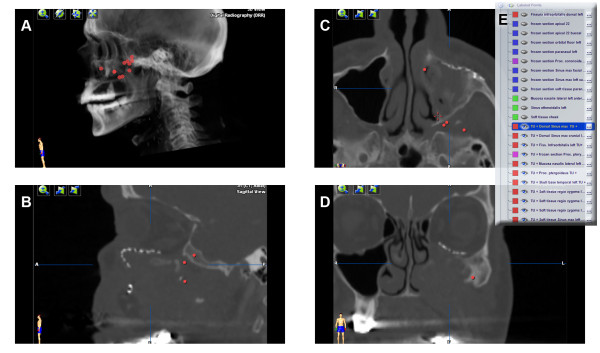
**Intra-operative marking of locations of frozen sections and surgical margins**. Only the tumor positive locations are labeled in red (A-D). List of all points (E).

The acquired intra-operative landmarks were postoperatively added to the virtual plan. After final release of the histological findings, the individual points were colour-coded according to their classification (benign/malignant). Visualization now allows for an optimized three-dimensional information about potential residual tumor (Figure 5).

### Export of the data set

The DICOM-format is a standard for transmitting information in medical imaging and was developed by the DICOM Standards Committee. With iPlan 4.0 beta (not released yet) the acquired point cloud could be written into the patient's DICOM data set. The authors selected only the points with tumor-positive histology. These points (voxel) were allocated with Houndsfield unit of 3500 H. This value is far out of the traditional highest range of around 3100 H and makes manipulation to the original DICOM data set obviously and fast segmentation of the point cloud by using threshold values possible.

### Import into the radiation therapy simulation platform

**Figure 6:**
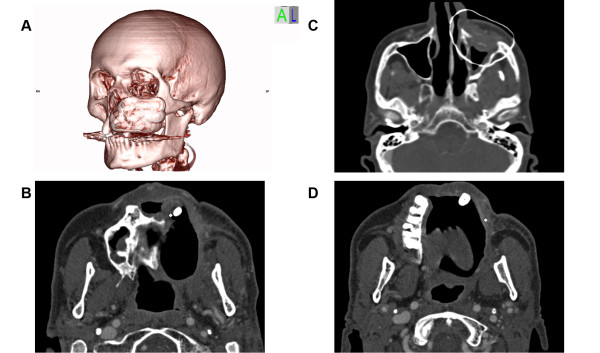
**Enhanced DICOM export into Osirix^®^**. DICOM with outlining of the augmented tumor resection area. (A) 3D reconstruction. (C) transversal view. Tumor positive points marked as single Voxel (B+D).

The thus enhanced DICOM data set (enDICOM) could be imported in any radiation therapy planning platform (Figure 6). The described enDICOM was routinely imported with the Oncentra MasterPlan (Version 3.3, Nucletron, Netherlands/USA) and superimposed with the post-operative CT scan. The well-defined high Houndsfield unit of the selected points facilitates segmentation and visualization of the additional intra-operative information and could be used to plan the geometric and radiological aspects of the therapy [11,12].

## Results and Discussion

The registration of patient data at the navigation system using screws as fiducial markers delivered a navigation accuracy with a mean deviation of 1.3 ± 0.6 mm in our cases. The novel method of intra-operative marking of specimen either frozen sections or surgical margins eases the storage and further use of intra-operative information. In the field of craniomaxillofacial surgery, indications for this technique are at the moment limited to tumor locations closed to hard tissue. But this technique is well used by tumors closed to liver, pancreas and adrenal. The more frequently head and neck malignancies affect the mandible, floor of the mouth and tongue. These locations do not allow for the described method. An adequate soft tissue navigation would be a necessary precondition. Currently assessing resection margins intraoperatively is possible by means of frozen sections. If positive they can be a guide to additional resection but when negative they add no information about the distance from the tumor of the margin [13]. So this computer-assisted surgical procedure might be a feasible solution. For the postoperative follow-up, it is a useful tool to correlate and transfer the outlined tumor boundaries into various image data sets to capture tumor recurrences or the result of adjuvant chemo- and radiotherapy to improve treatment outcome and quality of life [14].

## Conclusion

In the indicated patient's selection, the workflow, including preparation for Computer-assisted surgery (CAS), intra-operative navigation, gaining of tumor marking, and post-operative postprocessing of the acquired intra-operative individual data, was feasible. Augmented reality DICOM-data could be imported into the radiation therapy planning platform and could allow for optimizing of the treatment planning. The interface between surgeon and radiotherapist is significantly and verifiably simplified.

## List of abbreviations

3D: three-dimensional; CAPP: Computer-assisted preoperative planning; CAS: Computer assisted surgery; CBCT: cone beam computed tomography; CT: computed tomography; DICOM: Digital Imaging and Communications in Medicine; enDICOM: enhanced DICOM-data (augmented reality); MRI: magnetic resonance imaging; R0: complete removal of all tumor; R1: microscopic residuals of the tumor; R2: macroscopic residuals of the tumor; RMS: root mean square error.

## Competing interests

The authors declare that they have no competing interests.

## Authors' contributions

HE and MR contributed equally to this work. HE, MR, AM, AME, HK, CS, FT, DL, MRU and NCG conceived of the study and participated in its design and coordination. HE and MR made substantial contributions to conception and design of the manuscript as well as data acquisition. HE, MR, AME, NCG have been involved in drafting the manuscript. NCG was involved in revising the manuscript. All authors read and approved the final manuscript.

## Consent statement

Written informed consent was obtained from the patient for publication of this case report and accompanying images. A copy of the written consent is available for review by the Editor-in-Chief of this journal.

## Funding

The article processing charges are funded by the Deutsche Forschungsgemeinschaft (DFG), "Open Acess Publizieren".
